# The Liver and Glycogen: In Sickness and in Health

**DOI:** 10.3390/ijms24076133

**Published:** 2023-03-24

**Authors:** Gwyneth S. T. Soon, Michael Torbenson

**Affiliations:** 1 Department of Pathology, National University Hospital, Singapore 119074, Singapore; 2 Department of Laboratory Medicine and Pathology, Mayo Clinic, Rochester, MN 55905, USA

**Keywords:** glycogenic hepatopathy, Mauriac syndrome, hepatic glycogenosis, pseudoground glass, clear cell hepatocellular carcinoma, pathology

## Abstract

The liver is a major store of glycogen and is essential in maintaining systemic glucose homeostasis. In healthy individuals, glycogen synthesis and breakdown in the liver are tightly regulated. Abnormal glycogen metabolism results in prominent pathological changes in the liver, often manifesting as hepatic glycogenosis or glycogen inclusions. This can occur in genetic glycogen storage disease or acquired conditions with insulin dysregulation such as diabetes mellitus and non-alcoholic fatty liver disease or medication effects. Some primary hepatic tumors such as clear cell hepatocellular carcinoma also demonstrate excessive glycogen accumulation. This review provides an overview of the pathological manifestations and molecular mechanisms of liver diseases associated with abnormal glycogen accumulation.

## 1. Introduction

The liver plays a central role in carbohydrate metabolism. It is regarded as a “glucostat”, maintaining systemic glucose homeostasis through glucose uptake and storage post-prandially and glucose production and release during fasting. The physiologic regulation of hepatic glycogen synthesis, glycogenolysis, gluconeogenesis, glycolysis, and other enzymatic reactions is mediated through various mechanisms, including the balance of hormones such as insulin and glucagon, allosteric control by metabolites such as glucose, acetyl-coA, and glucose-6-phosphate, the availability of substrates, and the cellular redox state [1]. 

Dysregulation of glycogen metabolism in systemic conditions such as diabetes mellitus (DM), hepatic conditions such as non-alcoholic fatty liver disease (NAFLD), as well as genetic diseases (glycogen storage diseases) can lead to histological manifestations in the liver and cause significant hepatic dysfunction. When there is clinical uncertainty as to the cause of an abnormal liver enzyme profile, a liver biopsy can be helpful in guiding towards the correct diagnosis and excluding other diseases. The pathological changes, however, are often under-recognized or can be incorrectly attributed due to unfamiliarity with different patterns of injury as well as histological overlap between entities. 

In addition, glucose metabolism is often altered in cancer cells in order to support their rapid proliferation and expansion; this is part of metabolic reprogramming, a recognized hallmark of malignancy [2,3,4]. Most cancer cells demonstrate increased glucose uptake and glycolysis even in the presence of oxygen (aerobic glycolysis or the “Warburg effect” [5]). On the other hand, glycogen accumulation rather than glycogen depletion can be seen in some cancers, including primary hepatic tumors such as clear cell hepatocellular carcinomas (HCCs). The abundant glycogen content in these tumors results in a distinctive morphologic appearance that argues for their classification as a separate tumor subtype; nevertheless, the biological mechanisms underlying abnormal glycogen metabolism are complicated [6]. 

This review focuses on the histological manifestation of diseases associated with abnormal glycogen accumulation in the liver and briefly explores possible molecular mechanisms behind these changes.

## 2. Glycogen in the Normal Liver

The liver is a major store of glycogen. Glycogen is a branched polymer of glucose that is synthesized through the actions of glycogenin, glycogen synthase, and glycogen branching enzyme [7]. Glycogenin initiates the formation of a primary glucose chain and subsequently interacts with glycogen synthase to lengthen the glycogen-initiating particle. α-1,6-linkages are then added to the glycogen molecule by a glycogen-branching enzyme to create the final, spherical glycogen particle. 

Glycogen synthesis is regulated by phosphorylation/dephosphorylation and/or binding of allosteric ligands [8]. Glycogen degradation occurs through the actions of phosphorylase, a debranching enzyme, and acid α-glucosidase; the latter is predominantly lysosomal. Similar to glycogen synthase, the regulation of phosphorylase occurs through both phosphorylation and ligand binding [7].

The liver contains two types of glycogen: small β particles and larger α particles; the latter is a composite aggregate of β-particles [9]. α-particles release glucose more slowly than β-particles in the presence of degradative enzymes [10], thereby helping to maintain blood glucose levels during overnight fasting in diurnal mammals. Most glycogen is cytosolic, with up to 10% of glycogen in hepatocytes present within lysosomes [7]. 

**Figure 1:**
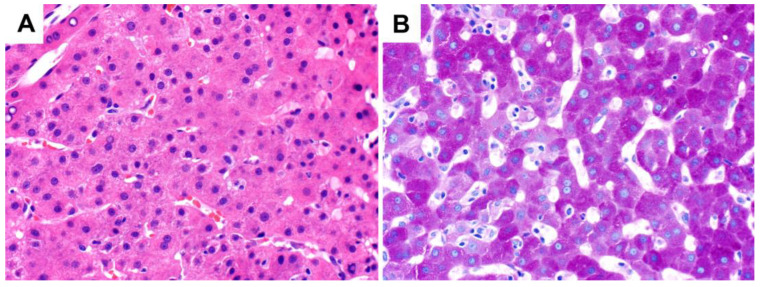
H&E and PAS staining characteristics in the normal liver (original magnification 400×): (**A**) H&E stain; (**B**) PAS stain.

In a normal state, all hepatocytes contain glycogen. The amount of glycogen is largely homogeneously distributed through out the liver lobule, although there can be zonal variations depending on the fasting or post-prandial state, corresponding to zonal variations in metabolic enzymatic activity [11]. On light microscopy with routine hematoxylin and eosin (H&E) staining, normal hepatocytes display abundant eosinophilic cytoplasm, and glycogen is not visible (Figure 1). Instead, the detection of glycogen requires the application of histochemical stains, classically the periodic acid–Schiff (PAS) and PAS with diastase digestion (PAS-D) stains [12]. PAS stains glycogen a bright magenta color. While other nonglycogen substances such as mucopolysaccharides will also react with the stain, only glycogen will be digested into water-soluble maltose by the subsequent application of diastase (α-amylase); thus, glycogen is subsequently washed out of the section, resulting in the absence of staining. Hence, the comparison of staining intensities with the PAS and PAS-D stains confirms the presence of glycogen. 

## 3. Pathological Conditions with Excessive Hepatic Glycogen Accumulation

Excessive glycogen accumulation within hepatocytes occurs in diseases caused or accompanied by the dysregulation of carbohydrate metabolism. In these pathological conditions, the excess glycogen is distinctly visible on H&E; the hepatocytes usually exhibit cytoplasmic pallor and rarefaction or may show cytoplasmic-glycogen-filled inclusion bodies. Some hepatocytes may also demonstrate glycogen-filled nuclear vacuoles without a delimiting membrane (“glycogenated nuclei”) [13]; these are frequently seen in patients with diabetes and obesity, although they can also be seen in other liver conditions such as Wilson’s disease [14]. The following subsections discuss the major conditions in which abnormal hepatic glycogen accumulation may be seen.

### 3.1. Glycogenic Hepatopathy

Glycogenic hepatopathy is a distinctive, reversible clinicopathological entity caused by excessive overloading of hepatocytes with glycogen, classically seen in patients with poorly controlled type 1 diabetes mellitus. While first documented as a component of Mauriac syndrome in 1930 [15], it was subsequently recognized that glycogen overloading of the liver can occur without the associated findings of growth failure, delayed puberty, and cushingoid features. Previously referred to variably as hepatic/liver glycogenosis [16,17], liver glycogen storage [18,19], or DM-associated glycogen storage hepatomegaly [20], the term “glycogenic hepatopathy” was coined in 2006 in the first paper to systematically describe the histological findings and has now gained widespread acceptance and usage [21]. 

#### 3.1.1. Clinical Findings and Pathogenesis

Glycogenic hepatopathy typically occurs in children and adults with marked or prolonged hyperglycemia, followed by exogenous administration of insulin in the setting of type 1 DM. Most patients have hepatomegaly and elevated serum aminotransferases, which improve after improved glycemic control [22]. Rarely, ascites may also be a presenting sign, but this too resolves with adequate control of blood sugar levels [21,23]. In the current era of modern insulin regimens and intensive glycemic monitoring, it is rare for patients to present with the full complement of Mauriac syndrome [21,24], although patients with glycogenic hepatopathy may have impaired growth compared to their peers [25].

**Figure 2:**
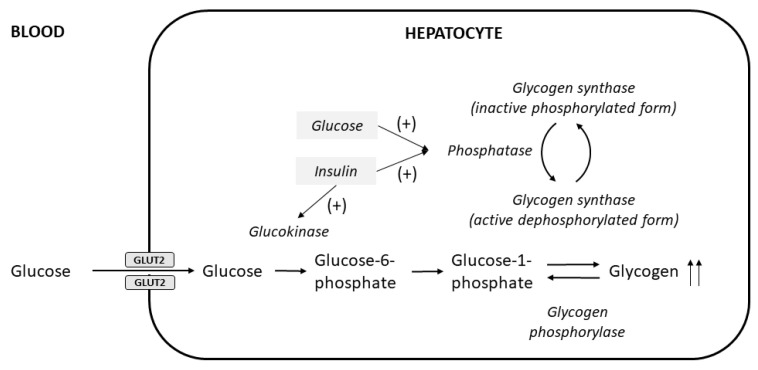
Pathogenesis of hepatocellular glycogen accumulation in the liver, highlighting the role of both elevated glucose levels and insulin treatment in glycogenic hepatopathy (modified from Munns et al. [26]). Glucose enters hepatocytes via facilitated diffusion through glucose transporter 2 (GLUT2) independent of insulin, where it is then phosphorylated to glucose-6-phosphate by glucokinase and subsequently converted to glycogen by glycogen synthase. Insulin activates glucokinase, and also acts together with elevated glucose levels to stimulate the phosphatase enzyme to produce the active dephosphorylated form of glycogen synthase [1].

The excessive glycogen accumulates in hepatocytes because of the insulin-independent passage of glucose into hepatocytes during the period of hyperglycemia, followed by insulin-mediated conversion into hepatic glycogen; both elevated glucose and insulin are required [26] (Figure 2). Patients who take excess insulin and are subsequently treated with glucose to correct hypoglycemia can also develop glycogenic hepatopathy [27]. Glycogenic hepatopathy has only rarely been reported in patients with type 2 DM [28]; the insulin resistance underlying type 2 DM typically leads to reduced hepatic glycogen synthesis and increased lipogenesis, manifesting predominantly as non-alcoholic fatty liver disease [29]. Nevertheless, glycogenic hepatocytes can also be present in these patients [30]. 

Apart from type 1 DM, glycogenic hepatopathy has also been described in other clinical conditions, such as following the administration of short-term high-dose steroid therapy [31], dumping syndrome [32], and anorexia nervosa [33,34]. The former two conditions result in a hyperglycemic–hyperinsulinemic state similar to that observed in type 1 DM patients. The mechanism behind excessive hepatic glycogen accumulation in anorexia nervosa is uncertain; it may be related to the body’s adaptation to starvation by sparing glucose utilization and stimulation of hepatic glycogenesis in order to prevent severe hypoglycemia and death [33,34].

A case–control study confirmed that patients with glycogenic hepatopathy had poorer glycemic control, as evidenced by a history of recurrent episodes of diabetic ketoacidosis and elevated HbA_1c_ levels [24]. However, it is still uncertain why only a small subset of patients with type 1 DM develop glycogenic hepatopathy. The excess glycogen accumulated in the hepatocytes appears to have a normal molecular structure, and the activity of enzymes glucose-6-phosphatase, phosphorylase acid maltase, amylo-1,6-glucosidase, and phosphoglucomutase appears largely preserved [35]. Although a heterozygous mutation in liver glycogen phosphorylase kinase was found in one patient with type 1 DM and Mauriac syndrome [36], this does not account for the vast majority of cases in which glycogenic hepatopathy is a reversible phenomenon. It may be that single nucleotide polymorphisms in genes involved in glycogen metabolism predispose certain individuals to developing or manifesting the disease [37].

#### 3.1.2. Pathological Findings

**Figure 3:**
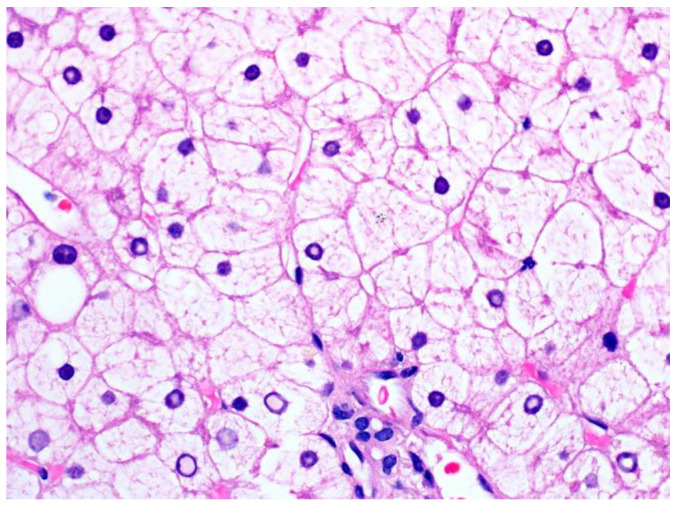
A liver biopsy from a patient with glycogenic hepatopathy demonstrating swollen hepatocytes with abundant clear cytoplasm due to glycogen accumulation (H&E stain; original magnification 600×). Occasional glycogenated nuclei can be seen.

Histologically, glycogenic hepatopathy is characterized by a striking diffuse hepatocellular change—the hepatocytes appear pale with cytoplasmic rarefaction and accentuated cell membranes, due to cytoplasmic accumulation of glycogen (Figure 3). PAS and PAS-D stains can be used to prove that the clearing of the cytoplasm is due to glycogen, but needs to be interpreted in conjunction with H&E and clinical findings, given that normal hepatocytes have abundant baseline amounts of glycogen. Glycogenated nuclei can also be present but are a nonspecific finding. The swollen hepatocytes compress the sinusoids, imparting a “paved” appearance. No disruption of the lobular architecture and no or only minimal inflammation is seen. Macrovesicular steatosis, usually mild to moderate, may also be present in some cases. 

Fibrosis is usually not a feature of glycogenic hepatopathy. Although occasional cases may have mild portal or pericellular fibrosis [21], only very rare cases of bridging fibrosis have been reported [25,38]. If there is significant fibrosis, other conditions such as steatohepatitis or diabetic hepatosclerosis should be excluded. Diabetic hepatosclerosis occurs in some patients with long-standing diabetes mellitus; it is characterized by extensive, dense perisinusoidal fibrosis in the absence of steatohepatitis [39]. Diabetic hepatosclerosis appears to be a microangiopathic disease of the liver and is often accompanied by hyaline arteriolosclerosis in the liver [40].

### 3.2. Glycogenosis in NAFLD 

**Figure 4:**
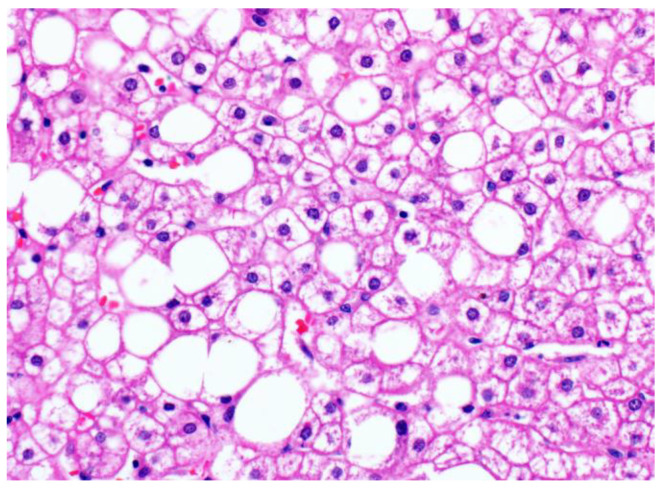
Patchy glycogenosis in NAFLD (original magnification 400×). The empty rounded spaces are large lipid droplets displacing the cell nuclei, while the glycogenated hepatocytes show cytoplasmic pallor but retain the central position of the nuclei and angular cell contours.

Recently, it has been recognized that excess accumulation of glycogen within hepatocytes can also be seen in the context of non-alcoholic fatty liver disease. In these cases, the dominant clinicopathological picture is not that of the typical glycogenic hepatopathy patient (who has type 1 DM and poor glycemic control, hepatomegaly and elevated transaminases); instead, patients are typically older, have type 2 DM and other features of metabolic syndrome, and steatohepatitis. These glycogenic hepatocytes can be seen in up to 54% of NAFLD cases [30]; the glycogenated hepatocytes occur focally, often in patches in the centrilobular zone (Figure 4). Even when glycogenated hepatocytes are present more extensively, the liver tissue in patients with NAFLD does not show the same diffuse striking hepatocellular changes characteristic of glycogenic hepatopathy. Individually, the enlarged, pale, swollen hepatocytes appear similar to those seen in glycogenic hepatopathy but lack the diffuse distribution of glycogenic hepatopathy. They may also be confused with ballooned hepatocytes, a form of hepatocyte injury with cytoskeletal alterations [41] that is used to distinguish steatohepatitis from simple steatosis [42]. Glycogenated nuclei may also be seen.

The presence of focal/patchy hepatocyte glycogenosis in a significant proportion of NAFLD patients underscores the complexity of glucose regulation. Insulin resistance, which is a major hallmark of NAFLD, impairs hepatic glycogen synthesis through dysregulation of glucokinase translocation from the nucleus to the cytoplasm, a rate-controlling step in insulin-stimulated hepatic glycogen synthesis [43]. Hepatocytes in patients with type 2 DM, characterized by insulin resistance, similarly have decreased glycogen content [44]. It is likely that dysregulation of the closely interrelated carbohydrate and lipid metabolic pathways in the liver can cause glycogenosis in this setting when insulin-mediated pathways are bypassed, leading to shunting of substrates from one path to another. Individuals at high risk of NAFLD who have a genetic variation in *PPP1R3B*, a hepatic glycogen metabolism regulatory protein involved in glycogen synthesis, have been reported to have reduced hepatic steatosis [45], possibly due to shunting of glucose to glycogen synthesis and reduction of de novo lipogenesis [46]. Indeed, glycogenosis in the liver biopsies of NAFLD patients appears to be associated with decreased steatosis [30]. 

### 3.3. Glycogen Storage Disease

Glycogen storage diseases (GSDs) are genetic disorders of glycogen metabolism, many of which present as an abnormal accumulation of glycogen or lipid in the liver. They are classified based on enzyme deficiency and the affected tissue (muscle and liver); the GSDs primarily affecting the liver are types 0, I, III, IV, VI, and IX [47]. The hepatic GSDs are generally characterized by hepatomegaly and fasting hypoglycemia, although the individual clinical and biochemical profiles will differ depending on the specific enzymatic defect [48] (Table 1). Readers may refer to recent comprehensive reviews on this topic for more details [48,49]. 

**Figure 5:**
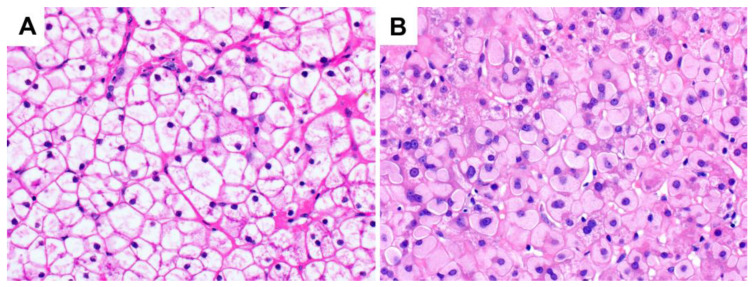
Glycogen storage disease (original magnification 400×): (**A**) type IX, with a similar histological appearance to glycogenic hepatopathy; (**B**) type IV, demonstrating hepatocellular ground-glass type cytoplasmic inclusions. These PAS-positive inclusions may be diastase-resistant as they comprise amylopectin-like material rather than typical glycogen. Chronic hepatitis B infection and drug-induced glycogen pseudoground-glass type changes can have a similar appearance and should be excluded.

The glycogenosis seen on liver biopsies in patients with hepatic GSD can look indistinguishable from that seen in glycogenic hepatopathy, although in some cases of GSD, there may be subtle but increased cytoplasmic clumping of glycogen, and in type IV there are distinctive ground-glass type inclusions (Figure 5). The clinical setting (the absence of poorly-controlled diabetes) is key to arriving at the correct diagnosis, with subsequent confirmation by additional biochemical assays or sequencing. 

Hepatic steatosis may also be present in patients with GSD. The dysregulation of liver glycogen metabolism often leads to compensatory increases in lipolysis from extrahepatic sources and mitochondrial fatty acid oxidation in GSD types associated with fasting ketotic hypoglycemia (0, III, VI, IX, and XI). In contrast, the hyperlipidemia seen in GSD type I results from the intracellular accumulation of glucose-6-phosphate, leading to increased glycolysis, increased production of acetyl-CoA, and increased lipogenesis [51].

## 4. Abnormal Hepatic Glycogen Accumulation in Miscellaneous Conditions 

Abnormal glycogen accumulations can sometimes also be seen in conditions not primarily driven by altered carbohydrate metabolism, such as urea cycle defects and drug-induced changes. 

### 4.1. Urea Cycle Defects 

The urea cycle occurs primarily in the liver and refers to a series of mitochondrial and cytosolic enzyme reactions that remove excess nitrogen from the body through the conversion of toxic ammonia into urea; urea is then excreted into the urine. Interestingly, foci of glycogenesis and glycogenated nuclei can be seen in patients with urea cycle defects, sometimes in a nodular manner; concomitant steatosis and varying degrees of fibrosis may also be present [52,53]. 

The pathogenesis of increased glycogen accumulation in patients with urea cycle defects remains to be determined. In one study, the activity of glucose-6-phosphatase, debranching enzyme, and phosphorylase appeared normal or only mildly abnormal. It was therefore suggested that the excess glycogen accumulation could be due to dietary treatment, wherein the high proportion of leucine could have stimulated excessive insulin secretion and glycogen synthesis [54]. More recently, however, a mouse model of arginosuccinate lyase deficiency showed impaired glycogenolysis due to reduction in hepatic glycogen phosphorylase activity, rather than increased glycogen synthesis; this appeared to cause the excess glycogen accumulation, possibly through post-translational modification of the enzyme [55]. 

### 4.2. Drug-Induced Pseudoground Glass Inclusions

**Figure 6:**
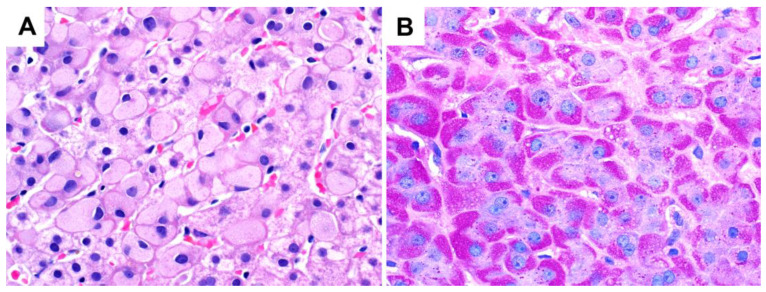
Drug-induced changes in the liver (original magnification 600×): (**A**) glycogen pseudoground glass changes in polypharmacy; (**B**) “two-tone” hepatocyte cytoplasmic appearance highlighted by PAS stain, possibly due to abnormal endoplasmic reticulum proliferation.

Glycogen pseudoground glass changes have been reported in patients taking numerous medications [56,57]. Pseudoground glass changes refer to large amphophilic-to-pale eosinophilic inclusion-like structures within hepatocytes that are single, PAS-positive, diastase sensitive, and often surrounded by a rim of normal cytoplasm (Figure 6A). On electron microscopy, these inclusions are composed of non-membrane-bound collections of abnormal glycogen along with some degenerating cell organelles [56,57,58]. They are also associated with several glycogen-associated proteins such as glycogenin-1, and it is thought that these inclusions result from disturbed glycogen metabolism induced by drugs binding to glycogenin [59]. Drugs may also result in hepatocytes having a “two-tone” cytoplasmic appearance that is highlighted by PAS stain (Figure 6B); in this histological pattern, the hepatocytes show normal cytoplasmic eosinophilia in one-half and a distinctive homogeneous gray appearance in the other half [60]. 

These drug-induced cytoplasmic changes may persist or resolve; their clinical significance, apart from being a histological mimic of other causes of pseudoground glass changes, is unclear at this time. 

## 5. Hepatic Masses with Abnormal Glycogen Accumulation

**Figure 7:**
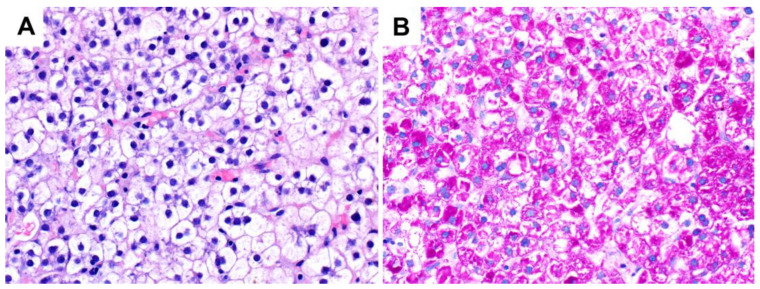
Clear cell hepatocellular carcinoma with abundant cytoplasmic glycogen (original magnification 400×): (**A**) H&E; (**B**) PAS stain.

Liver masses can have abnormal glycogen accumulation which leads to a clear cell appearance. These include cases of hepatic adenomas (particularly sonic hedgehog hepatic adenoma [61]) and clear cell hepatocellular carcinomas (Figure 7). A rare case of hepatocellular carcinoma with glycogen ground-glass changes has also been reported; in this case, the tumor but not the non-tumor tissue showed complete absence of glucose-6-phosphatase activity, suggesting that the tumor cells had acquired enzyme deficiency, leading to abnormal glycogen accumulation [62]. Some bile duct adenomas and intrahepatic cholangiocarcinomas can exhibit a clear cell morphology, but in these cases, the cytoplasmic pallor may be due to mucin or lipid vacuoles, respectively, rather than abnormal glycogen accumulation [63,64].

Metabolic reprogramming, the alteration of a cancer cell’s metabolism, is important in carcinogenesis; it facilitates cell growth, proliferation, and other cancer-associated hallmarks [2,3,4]. Although it was initially thought that many of the cellular metabolic changes in cancer cells were due to damaged mitochondria or a secondary phenomenon of cell proliferation/survival signals, it is now recognized that altered metabolism can result from active reprogramming through the actions of oncogenes, tumor suppressors, and metabolic enzymes, as well as an adaptation to other factors both intrinsic and extrinsic to the cancer cell, leading to remarkable metabolic heterogeneity even within the same tumor [3,4,65]. Furthermore, these metabolic phenotypes can evolve during tumorigenesis and cancer progression, depending on the selective pressures exerted [3,65].

The metabolic reprogramming underlying the accumulation of intracytoplasmic glycogen that typifies clear cell HCC has been challenging to study. There is currently no uniform morphologic definition for clear cell HCC; neither has any clear genetic correlate been identified [66]. One study found *isocitrate dehydrogenase 1* (*IDH1)* R132C mutations in 5 out of 20 clear cell HCCs (25%) by pyrosequencing; this mutation was absent in 28 HCCs with other morphologies in their study, although the authors did find 3 out of 193 HCCs (1.6%) from The Cancer Genome Atlas with non-clear cell morphologies that also had *IDH1* mutations [67]. Isocitrate dehydrogenase 1 is a NADP^+^-dependent enzyme that is highly expressed in the liver [68] and catalyzes the oxidative decarboxylation of isocitrate to α-ketoglutarate [69]. In normal cells, the NADPH produced through this enzymatic reaction is thought to participate in cellular defense against oxidative stress [70] and also plays a major role in lipid metabolism [71]. More recently, IDH1 has also been reported to be important in hepatic amino acid utilization and gluconeogenesis. On the other hand, no apparent change in liver glycogen content in a mouse model with IDH1 deficiency was seen [72]. 

Increased *IDH1* gene expression has been shown to correlate with mRNA levels of canonical sterol regulatory element binding protein (SREBP) gene targets in several different human cancers, including HCC [73]. The SREBP transcription factors are major drivers of lipid synthesis [74]. Of note, early ultrastructural studies reported accumulation of both glycogen and lipid within the neoplastic hepatocytes of clear cell HCC [75,76,77]. It may be that cases of clear cell HCC with *IDH1* mutations demonstrate a greater proportion of intracytoplasmic lipid rather than glycogen to impart the optically clear cytoplasmic appearance. Certainly, some clear cell HCCs appear to have foci of microvesicular or even macrovesicular steatosis that is evident on H&E. Some degree of shunting of substrates across the closely interrelated glucose and lipid metabolic pathways is not unexpected. Contrary to what is observed in glycogenosis in NAFLD patients, however, some studies have noted an increase, rather than decrease, in the incidence of fatty change within HCC with an increasing proportion of clear cells [76,78]. While the presence of fatty change may be due to other confounding or unrelated factors such as tumor size [78], this association between glycogen and lipid accumulation nevertheless underscores the complexity of the various metabolic alterations that can occur in liver cancer cells. 

The metabolic alterations and genetic basis underlying cases of clear cell HCC with predominant glycogen accumulation and little/no steatosis may be different from those tumors with some fat. It is thought that, at least in some settings, glycogen synthesis and accumulation is an adaptive response to hypoxia through the effects of hypoxia-inducible factor (HIF), in anticipation of an unfavorable environment deficient in glucose during which rapid mobilization of these glycogen stores can then occur [79]. In clear cell renal cell carcinomas, another tumor characterized by abundant intracellular glycogen accumulation, glycogen accumulation is driven by loss-of-function mutations in the von Hippel Lindau (vHL) tumor suppressor gene that results in constitutive activation of HIF-α proteins [80]. It is uncertain whether the HIF pathway is similarly activated in clear cell HCCs, which lack vHL mutations. 

In clear cell renal cell carcinomas, glycogen synthase 1 (GYS1) was found to be overexpressed and promoted tumor growth [81]. Increased expression of GYS2, the isoform found in the liver, has been associated with increased glycogen content in HCC; however, in contrast to clear cell renal cell carcinoma, GYS2 overexpression correlated with decreased cell proliferation, possibly through activation of the p53 signaling pathway by competitive binding to MDM2 to inhibit the ubiquitination of p53 [82]. The same study also found that the glycogen content in HCC demonstrated a positive correlation with carbonic anhydrase 9 (CA9) and a negative correlation with liver glycogen phosphorylase (PYGL), suggesting that other factors were involved in regulating the glycogen content in HCC. While this study provides mechanistic insights into deregulation of glycogen metabolism in HCC in general, it did not specifically study clear cell HCC, and the majority of the cases were from hepatitis B patients. HBx, an oncoprotein encoded by the hepatitis B virus, was thought to be involved in suppressing GYS2 expression. In other studies on HCC investigating correlates between tumor morphology and transcriptomic profiles, HCCs with a clear cell population were associated with transcriptomic subgroups characterized by cell cycle activation and proliferation [83,84], and dysregulation of several genes involved in glycolysis, gluconeogenesis, and fatty acid synthesis such as glucose phosphate isomerase and phosphoenolpyruvate carboxykinase 2 [84]. 

The metabolic phenotypes of tumors may change as cancers progress from preneoplastic lesions to established malignancies. Recent work has suggested that glycogen accumulation is important in liver tumor initiation, possibly through sequestration of Hippo kinases Mst1/2 and indirect activation of the oncoprotein Yap [85]; in this context, glycogen performs a signaling role beyond its usual function as a carbon-store. However, characterizing metabolic anomalies in premalignant or early lesions is challenging for two reasons: (1) these lesions may not be well characterized as precursor lesions or often escape clinical attention, and (2) their metabolism is probably influenced to some extent by the body’s systemic metabolism, such as the presence of obesity and diabetes [65]. Indeed, there are some early well-differentiated hepatocellular carcinomas with patchy rather than diffuse clear cell change, suggesting that glycogen accumulation may be a secondary rather than primary phenomenon in some cases. 

It is likely that the clear cell subtype of HCC (as currently defined by pure morphology) represents several biologically different entities, with overlapping morphology but differing underlying molecular mechanisms. It remains to be determined whether the accumulated glycogen in clear cell HCC is truly essential for tumor initiation and/or growth [85] or a dispensable consequence of a separate pathway [86].

## 6. Conclusions

Glycogen in hepatocytes is increasingly understood as not just a simple inert macromolecule but a complex molecule with important ramifications on metabolic pathways. The liver, as the main site of glycogen synthesis, storage, and lysis, manifests many of the key pathological changes associated with abnormal glycogen metabolism. Important liver diseases that result from glycogen accumulation include genetic glycogen storage diseases and acquired diseases resulting from insulin dysregulation and/or medication effects. Some primary hepatic tumors may also manifest prominent glycogen accumulation. Future work focusing on the mechanisms behind conditions with dysregulated glycogen metabolic pathways will lead to a better understanding of the significance of these changes on health and disease.

## Figures and Tables

**Table 1:** Glycogen storage disorders that primarily affect the liver [48,50].

Types	Gene Mutation	Defective Protein	Clinical Manifestations	Main Liver Findings
0	*GYS2*	Glycogen synthetase	Hypoglycemia	Steatosis
Ia	*G6PC*	Glucose-6-phosphatase	Short stature, hepatomegaly, hypoglycemia, and increased triglycerides, lactate, and uric acid	Steatosis Glycogenosis
Ib	*SLC37A4*	Glucose-6-phosphate translocase	Similar to type Ia with additional neutropenia, infections, and poor wound healing	Steatosis Glycogenosis
III	*AGL*	Debranching enzyme and variants	Short stature, hepatomegaly, hypoglycemia, hyperlipidemia, and cardiomyopathy	Steatosis Glycogenosis
IV	*GBE1*	1,4-alpha-glucan branching enzyme 1	Progressive form: short stature, hepatomegaly, splenomegaly, cirrhosis, and late-onset hypoglycemia Non-progressive form: hepatomegaly	Hepatocyte inclusions resembling ground glass changes
VI	*PYGL*	Liver phosphorylase	Short stature, hepatomegaly, and hypoglycemia	Steatosis Glycogenosis
IX	*PHKA1, PHKA2, PHKB, PHKG2*	Phosphorylase kinase and variants	Short stature, hepatomegaly, hypoglycemia, hyperlipidemia, and myopathy	Glycogenosis

## Data Availability

No new data were created or analyzed in this study. Data sharing is not applicable to this article.

## References

[1] Petersen M.C., Vatner D.F., Shulman G.I. (2017). Regulation of Hepatic Glucose Metabolism in Health and Disease. Nat. Rev. Endocrinol..

[2] Hanahan D., Weinberg R.A. (2011). Hallmarks of Cancer: The next Generation. Cell.

[3] Yoshida G.J. (2015). Metabolic Reprogramming: The Emerging Concept and Associated Therapeutic Strategies. J. Exp. Clin. Cancer Res..

[4] Ward P.S., Thompson C.B. (2012). Metabolic Reprogramming: A Cancer Hallmark Even Warburg Did Not Anticipate. Cancer Cell.

[5] Warburg O., Wind F., Negelein E. (1927). The Metabolism of Tumors in The Body. J. Gen. Physiol..

[6] Khan T., Sullivan M.A., Gunter J.H., Kryza T., Lyons N., He Y., Hooper J.D. (2020). Revisiting Glycogen in Cancer: A Conspicuous and Targetable Enabler of Malignant Transformation. Front. Oncol..

[7] Roach P.J. (2002). Glycogen and Its Metabolism. Curr. Mol. Med..

[8] Marr L., Biswas D., Daly L.A., Browning C., Vial S.C.M., Maskell D.P., Hudson C., Bertrand J.A., Pollard J., Ranson N.A. (2022). Mechanism of Glycogen Synthase Inactivation and Interaction with Glycogenin. Nat. Commun..

[9] Tan X., Sullivan M.A., Nada S.S., Deng B., Schulz B.L., Gilbert R.G. (2018). Proteomic Investigation of the Binding Agent between Liver Glycogen β Particles. ACS Omega.

[10] Jiang X., Zhang P., Li S., Tan X., Hu Z., Deng B., Wang K., Li C., Sullivan M.A., Li E. (2016). Molecular-Size Dependence of Glycogen Enzymatic Degradation and Its Importance for Diabetes. Eur. Polym. J..

[11] Jungermann K. (1987). Metabolic Zonation of Liver Parenchyma: Significance for the Regulation of Glycogen Metabolism, Gluconeogenesis, and Glycolysis. Diabetes Metab. Rev..

[12] Layton C., Bancroft J.D., Suvarna S.K., Layton C., Bancroft J.D. (2019). Carbohydrates. Bancroft’s Theory and Practice of Histological Techniques.

[13] Schwertheim S., Kälsch J., Jastrow H., Schaefer C.M., Theurer S., Ting S., Canbay A., Wedemeyer H., Schmid K.W., Baba H.A. (2020). Characterization of Two Types of Intranuclear Hepatocellular Inclusions in NAFLD. Sci. Rep..

[14] Abraham S., Furth E.E. (1994). Receiver Operating Characteristic Analysis of Glycogenated Nuclei in Liver Biopsy Specimens: Quantitative Evaluation of Their Relationship with Diabetes and Obesity. Hum. Pathol..

[15] Mauriac P. (1930). Gros Ventre, Hepatomegalie, Troubles de Las Croissance Chez Les Enfants Diabetiques Traits Depuis Plusieurs Annes Par l’insuline. Gax. Hebd. Med. Bordx..

[16] Carcione L., Lombardo F., Messina M.F., Rosano M., De Luca F. (2003). Liver Glycogenosis as Early Manifestation in Type 1 Diabetes Mellitus. Diabetes Nutr. Metab..

[17] Chatila R., West A.B. (1996). Hepatomegaly and Abnormal Liver Tests Due to Glycogenosis in Adults with Diabetes. Medicine.

[18] Evans R.W., Littler T.R., Pemberton H.S. (1955). Glycogen Storage in the Liver in Diabetes Mellitus. J. Clin. Pathol..

[19] Torres M., López D. (2001). Liver Glycogen Storage Associated with Uncontrolled Type 1 Diabetes Mellitus. J. Hepatol..

[20] Nakamuta M., Ohashi M., Goto K., Tanabe Y., Hiroshige K., Nawata H. (1993). Diabetes Mellitus-Associated Glycogen Storage Hepatomegaly: Report of a Case and Review of the Japanese Literature. Fukuoka Igaku Zasshi.

[21] Torbenson M., Chen Y.-Y., Brunt E., Cummings O.W., Gottfried M., Jakate S., Liu Y.-C., Yeh M.M., Ferrell L. (2006). Glycogenic Hepatopathy: An Underrecognized Hepatic Complication of Diabetes Mellitus. Am. J. Surg. Pathol..

[22] Haffar S., Izzy M., Habib H., Sugihara T., Li D.K., Sharma A., Wang Z., Murad M.H., Watt K.D., Bazerbachi F. (2021). Liver Chemistries in Glycogenic Hepatopathy Associated with Type 1 Diabetes Mellitus: A Systematic Review and Pooled Analysis. Liver Int..

[23] Bronstein H.D., Kantrowitz P.A., Schaffner F. (1959). Marked Enlargement of the Liver and Transient Ascites Associated with the Treatment of Diabetic Acidosis. N. Engl. J. Med..

[24] Mukewar S., Sharma A., Lackore K.A., Enders F.T., Torbenson M.S., Kamath P.S., Roberts L.R., Kudva Y.C. (2017). Clinical, Biochemical, and Histopathology Features of Patients With Glycogenic Hepatopathy. Clin. Gastroenterol. Hepatol..

[25] Fitzpatrick E., Cotoi C., Quaglia A., Sakellariou S., Ford-Adams M.E., Hadzic N. (2014). Hepatopathy of Mauriac Syndrome: A Retrospective Review from a Tertiary Liver Centre. Arch. Dis. Child..

[26] Munns C.F., McCrossin R.B., Thomsett M.J., Batch J. (2000). Hepatic Glycogenosis: Reversible Hepatomegaly in Type 1 Diabetes. J. Paediatr. Child Health.

[27] Tsujimoto T., Takano M., Nishiofuku M., Yoshiji H., Matsumura Y., Kuriyama S., Uemura M., Okamoto S., Fukui H. (2006). Rapid Onset of Glycogen Storage Hepatomegaly in a Type-2 Diabetic Patient after a Massive Dose of Long-Acting Insulin and Large Doses of Glucose. Intern. Med..

[28] Umpaichitra V. (2016). Unusual Glycogenic Hepatopathy Causing Abnormal Liver Enzymes in a Morbidly Obese Adolescent with Well-Controlled Type 2 Diabetes: Resolved after A1c Was Normalized by Metformin. Clin. Obes..

[29] Jiang S., Young J.L., Wang K., Qian Y., Cai L. (2020). Diabetic-induced Alterations in Hepatic Glucose and Lipid Metabolism: The Role of Type 1 and Type 2 Diabetes Mellitus (Review). Mol. Med. Rep..

[30] Allende D.S., Gawrieh S., Cummings O.W., Belt P., Wilson L., Van Natta M., Behling C.A., Carpenter D., Gill R.M., Kleiner D.E. (2021). Glycogenosis Is Common in Nonalcoholic Fatty Liver Disease and Is Independently Associated with Ballooning, but Lower Steatosis and Lower Fibrosis. Liver Int..

[31] Iancu T.C., Shiloh H., Dembo L. (1986). Hepatomegaly Following Short-Term High-Dose Steroid Therapy. J. Pediatr. Gastroenterol. Nutr..

[32] Resnick J.M., Zador I., Fish D.L. (2011). Dumping Syndrome, a Cause of Acquired Glycogenic Hepatopathy. Pediatr. Dev. Pathol..

[33] Kransdorf L.N., Millstine D., Smith M.L., Aqel B.A. (2016). Hepatic Glycogen Deposition in a Patient with Anorexia Nervosa and Persistently Abnormal Transaminase Levels. Clin. Res. Hepatol. Gastroenterol..

[34] Komuta M., Harada M., Ueno T., Uchimura Y., Inada C., Mitsuyama K., Sakisaka S., Sata M., Tanikawa K. (1998). Unusual Accumulation of Glycogen in Liver Parenchymal Cells in a Patient with Anorexia Nervosa. Intern. Med..

[35] Manderson W.G., McKiddie M.T., Manners D.J., Stark J.R. (1968). Liver Glycogen Accumulation in Unstable Diabetes. Diabetes.

[36] MacDonald M.J., Hasan N.M., Ansari I.-U.H., Longacre M.J., Kendrick M.A., Stoker S.W. (2016). Discovery of a Genetic Metabolic Cause for Mauriac Syndrome in Type 1 Diabetes. Diabetes.

[37] Tomihira M., Kawasaki E., Nakajima H., Imamura Y., Sato Y., Sata M., Kage M., Sugie H., Nunoi K. (2004). Intermittent and Recurrent Hepatomegaly Due to Glycogen Storage in a Patient with Type 1 Diabetes: Genetic Analysis of the Liver Glycogen Phosphorylase Gene (PYGL). Diabetes Res. Clin. Pract..

[38] Sherigar J.M., Darouichi Y., Guss D., Mohanty S.R. (2018). An Unusual Presentation of Glycogenic Hepatopathy with Bridging Fibrosis. ACG Case Rep. J.

[39] Harrison S.A., Brunt E.M., Goodman Z.D., Di Bisceglie A.M. (2006). Diabetic Hepatosclerosis: Diabetic Microangiopathy of the Liver. Arch. Pathol. Lab. Med..

[40] Balakrishnan M., Garcia-Tsao G., Deng Y., Ciarleglio M., Jain D. (2015). Hepatic Arteriolosclerosis: A Small-Vessel Complication of Diabetes and Hypertension. Am. J. Surg. Pathol..

[41] Lackner C., Gogg-Kamerer M., Zatloukal K., Stumptner C., Brunt E.M., Denk H. (2008). Ballooned Hepatocytes in Steatohepatitis: The Value of Keratin Immunohistochemistry for Diagnosis. J. Hepatol..

[42] Gramlich T., Kleiner D.E., McCullough A.J., Matteoni C.A., Boparai N., Younossi Z.M. (2004). Pathologic Features Associated with Fibrosis in Nonalcoholic Fatty Liver Disease. Hum. Pathol..

[43] Nozaki Y., Petersen M.C., Zhang D., Vatner D.F., Perry R.J., Abulizi A., Haedersdal S., Zhang X.-M., Butrico G.M., Samuel V.T. (2020). Metabolic Control Analysis of Hepatic Glycogen Synthesis In Vivo. Proc. Natl. Acad. Sci. USA.

[44] Krssak M., Brehm A., Bernroider E., Anderwald C., Nowotny P., Dalla Man C., Cobelli C., Cline G.W., Shulman G.I., Waldhäusl W. (2004). Alterations in Postprandial Hepatic Glycogen Metabolism in Type 2 Diabetes. Diabetes.

[45] Dongiovanni P., Meroni M., Mancina R.M., Baselli G., Rametta R., Pelusi S., Männistö V., Fracanzani A.L., Badiali S., Miele L. (2018). Protein Phosphatase 1 Regulatory Subunit 3B Gene Variation Protects against Hepatic Fat Accumulation and Fibrosis in Individuals at High Risk of Nonalcoholic Fatty Liver Disease. Hepatol. Commun..

[46] Stender S., Smagris E., Lauridsen B.K., Kofoed K.F., Nordestgaard B.G., Tybjaerg-Hansen A., Pennacchio L.A., Dickel D.E., Cohen J.C., Hobbs H.H. (2018). Relationship between Genetic Variation at PPP1R3B and Levels of Liver Glycogen and Triglyceride. Hepatology.

[47] Ozen H. (2007). Glycogen Storage Diseases: New Perspectives. World J. Gastroenterol..

[48] Wright T.L.F., Umaña L.A., Ramirez C.M. (2022). Update on Glycogen Storage Disease: Primary Hepatic Involvement. Curr. Opin. Pediatr..

[49] Massese M., Tagliaferri F., Dionisi-Vici C., Maiorana A. (2022). Glycogen Storage Diseases with Liver Involvement: A Literature Review of GSD Type 0, IV, VI, IX and XI. Orphanet. J. Rare Dis..

[50] Torbenson M.S. (2022). Genetic Diseases of the Liver. Biopsy Interpretation of the Liver.

[51] Derks T.G.J., van Rijn M. (2015). Lipids in Hepatic Glycogen Storage Diseases: Pathophysiology, Monitoring of Dietary Management and Future Directions. J. Inherit. Metab. Dis..

[52] Badizadegan K., Perez-Atayde A.R. (1997). Focal Glycogenosis of the Liver in Disorders of Ureagenesis: Its Occurrence and Diagnostic Significance. Hepatology.

[53] Yaplito-Lee J., Chow C.-W., Boneh A. (2013). Histopathological Findings in Livers of Patients with Urea Cycle Disorders. Mol. Genet. Metab..

[54] Miles L., Heubi J.E., Bove K.E. (2005). Hepatocyte Glycogen Accumulation in Patients Undergoing Dietary Management of Urea Cycle Defects Mimics Storage Disease. J. Pediatr. Gastroenterol. Nutr..

[55] Burrage L.C., Madan S., Li X., Ali S., Mohammad M., Stroup B.M., Jiang M.-M., Cela R., Bertin T., Jin Z. (2020). Chronic Liver Disease and Impaired Hepatic Glycogen Metabolism in Argininosuccinate Lyase Deficiency. JCI Insight.

[56] Wisell J., Boitnott J., Haas M., Anders R.A., Hart J., Lewis J.T., Abraham S.C., Torbenson M. (2006). Glycogen Pseudoground Glass Change in Hepatocytes. Am. J. Surg. Pathol..

[57] Lefkowitch J.H., Lobritto S.J., Brown R.S., Emond J.C., Schilsky M.L., Rosenthal L.A., George D.M., Cairo M.S. (2006). Ground-Glass, Polyglucosan-like Hepatocellular Inclusions: A “New” Diagnostic Entity. Gastroenterology.

[58] Lu H.-C., González I.A., Byrnes K. (2021). Ground-Glass Hepatocellular Inclusions Are Associated with Polypharmacy. Ann. Diagn. Pathol..

[59] Callea F., Francalanci P., Grimaldi C., Camassei F.D., Devito R., Facchetti F., Alaggio R., Bellacchio E. (2022). Pathomorphogenesis of Glycogen-Ground Glass Hepatocytic Inclusions (Polyglucosan Bodies) in Children after Liver Transplantation. Int. J. Mol. Sci..

[60] Torbenson M.S. (2022). Drug-Induced Liver Injury. Biopsy Interpretation of the Liver.

[61] Sala M., Gonzales D., Leste-Lasserre T., Dugot-Senant N., Paradis V., Di Tommaso S., Dupuy J.-W., Pitard V., Dourthe C., Sciarra A. (2020). ASS1 Overexpression: A Hallmark of Sonic Hedgehog Hepatocellular Adenomas; Recommendations for Clinical Practice. Hepatol. Commun..

[62] Callea F., Giovannoni I., Stefanelli M., Villanacci V., Lorini G., Francalanci P. (2012). Glycogenotic Hepatocellular Carcinoma with Glycogen-Ground-Glass Hepatocytes: Histological, Histochemical and Microbiochemical Characterization of the Novel Variant. Histopathology.

[63] Haas S., Gütgemann I., Wolff M., Fischer H.-P. (2007). Intrahepatic Clear Cell Cholangiocarcinoma: Immunohistochemical Aspects in a Very Rare Type of Cholangiocarcinoma. Am. J. Surg. Pathol..

[64] Wu W.W., Gu M., Lu D. (2014). Cytopathologic, Histopathologic, and Immunohistochemical Features of Intrahepatic Clear Cell Bile Duct Adenoma: A Case Report and Review of the Literature. Case Rep. Pathol..

[65] Faubert B., Solmonson A., DeBerardinis R.J. (2020). Metabolic Reprogramming and Cancer Progression. Science.

[66] Torbenson M.S., Park Y.N., Sakamoto M., Roncalli M., Ng I. (2019). Hepatocellular Carcinoma. WHO Classification of Tumours Editorial Board. Digestive System Tumours.

[67] Lee J.H., Shin D.H., Park W.Y., Shin N., Kim A., Lee H.J., Kim Y.K., Choi K.U., Kim J.Y., Yang Y.I. (2017). IDH1 R132C Mutation Is Detected in Clear Cell Hepatocellular Carcinoma by Pyrosequencing. World J. Surg. Oncol..

[68] Fagerberg L., Hallström B.M., Oksvold P., Kampf C., Djureinovic D., Odeberg J., Habuka M., Tahmasebpoor S., Danielsson A., Edlund K. (2014). Analysis of the Human Tissue-Specific Expression by Genome-Wide Integration of Transcriptomics and Antibody-Based Proteomics. Mol. Cell Proteom..

[69] Nekrutenko A., Hillis D.M., Patton J.C., Bradley R.D., Baker R.J. (1998). Cytosolic Isocitrate Dehydrogenase in Humans, Mice, and Voles and Phylogenetic Analysis of the Enzyme Family. Mol. Biol. Evol..

[70] Minard K.I., McAlister-Henn L. (1999). Dependence of Peroxisomal Beta-Oxidation on Cytosolic Sources of NADPH. J. Biol. Chem..

[71] Koh H.-J., Lee S.-M., Son B.-G., Lee S.-H., Ryoo Z.Y., Chang K.-T., Park J.-W., Park D.-C., Song B.J., Veech R.L. (2004). Cytosolic NADP^+^-Dependent Isocitrate Dehydrogenase Plays a Key Role in Lipid Metabolism. J. Biol. Chem..

[72] Ye J., Gu Y., Zhang F., Zhao Y., Yuan Y., Hao Z., Sheng Y., Li W.Y., Wakeham A., Cairns R.A. (2017). IDH1 Deficiency Attenuates Gluconeogenesis in Mouse Liver by Impairing Amino Acid Utilization. Proc. Natl. Acad. Sci. USA.

[73] Ricoult S.J.H., Dibble C.C., Asara J.M., Manning B.D. (2016). Sterol Regulatory Element Binding Protein Regulates the Expression and Metabolic Functions of Wild-Type and Oncogenic IDH1. Mol. Cell Biol..

[74] Horton J.D., Goldstein J.L., Brown M.S. (2002). SREBPs: Activators of the Complete Program of Cholesterol and Fatty Acid Synthesis in the Liver. J. Clin. Investig..

[75] Wu P.C., Lai C.L., Lam K.C., Lok A.S., Lin H.J. (1983). Clear Cell Carcinoma of Liver. An Ultrastructural Study. Cancer.

[76] Yang S.H., Watanabe J., Nakashima O., Kojiro M. (1996). Clinicopathologic Study on Clear Cell Hepatocellular Carcinoma. Pathol. Int..

[77] Audisio R.A., Bombelli L., Lombardi L., Andreola S. (1987). A Clinico-Pathologic Study of Clear-Cell Hepatocellular Carcinoma. Tumori.

[78] Kiyomatsu K. (1989). Pathomorphologic study on hepatocellular carcinoma (HCC). A study of fatty change in HCC. Acta Hepatol. Jpn..

[79] Pelletier J., Bellot G., Gounon P., Lacas-Gervais S., Pouysségur J., Mazure N.M. (2012). Glycogen Synthesis Is Induced in Hypoxia by the Hypoxia-Inducible Factor and Promotes Cancer Cell Survival. Front. Oncol..

[80] Shen C., Kaelin W.G. (2013). The VHL/HIF Axis in Clear Cell Renal Carcinoma. Semin. Cancer Biol..

[81] Chen S.-L., Huang Q.-S., Huang Y.-H., Yang X., Yang M.-M., He Y.-F., Cao Y., Guan X.-Y., Yun J.-P. (2020). GYS1 Induces Glycogen Accumulation and Promotes Tumor Progression via the NF-ΚB Pathway in Clear Cell Renal Carcinoma. Theranostics.

[82] Chen S.-L., Zhang C.Z., Liu L.-L., Lu S.-X., Pan Y.-H., Wang C.-H., He Y.-F., Lin C.-S., Yang X., Xie D. (2019). A GYS2/P53 Negative Feedback Loop Restricts Tumor Growth in HBV-Related Hepatocellular Carcinoma. Cancer Res..

[83] Tan P.S., Nakagawa S., Goossens N., Venkatesh A., Huang T., Ward S.C., Sun X., Song W.-M., Koh A., Canasto-Chibuque C. (2016). Clinicopathological Indices to Predict Hepatocellular Carcinoma Molecular Classification. Liver Int..

[84] Calderaro J., Couchy G., Imbeaud S., Amaddeo G., Letouzé E., Blanc J.-F., Laurent C., Hajji Y., Azoulay D., Bioulac-Sage P. (2017). Histological Subtypes of Hepatocellular Carcinoma Are Related to Gene Mutations and Molecular Tumour Classification. J. Hepatol..

[85] Liu Q., Li J., Zhang W., Xiao C., Zhang S., Nian C., Li J., Su D., Chen L., Zhao Q. (2021). Glycogen Accumulation and Phase Separation Drives Liver Tumor Initiation. Cell.

[86] Xie H., Song J., Godfrey J., Riscal R., Skuli N., Nissim I., Simon M.C. (2021). Glycogen Metabolism Is Dispensable for Tumour Progression in Clear Cell Renal Cell Carcinoma. Nat. Metab..

